# Factors associated with extubation failure in an intensive care unit: a case-control study

**DOI:** 10.1590/1518-8345.6224.3864

**Published:** 2023-03-27

**Authors:** Ana Beatriz Braga Arcanjo, Lúcia Marinilza Beccaria

**Affiliations:** 1 Fundação Faculdade de Medicina de São José do Rio Preto, UTI 5 Bloco A, São José do Rio Preto, SP, Brazil.; 2 Faculdade de Medicina de São José do Rio Preto, Departamento de Enfermagem Especializada, São José do Rio Preto, SP, Brazil.

**Keywords:** Patient, Extubation, Risk Factors, Treatment Failure, Artificial Respiration, Intensive Care Unit, Paciente, Extubação, Fatores de Risco, Falha de Tratamento, Respiração Artificial, Unidade de Terapia Intensiva, Paciente, Extubación Traqueal, Factores de Riesgo, Insuficiencia del Tratamiento, Respiración Artificial, Unidad de Cuidado Intensivo

## Abstract

**Objective:**

to investigate the factors associated with extubation failure of patients in the intensive care unit.

**Method:**

unpaired, longitudinal, retrospective and quantitative case-control with the participation of 480 patients through clinical parameters for ventilator weaning. Data were analyzed by: Fisher’s exact test or the chi-square test; unpaired two-tailed Student’s t test; and Mann-Whitney test. Significant P values lower than or equal to 0.05 were admitted.

**Results:**

of the patients, 415 (86.5%) were successful and 65 (13.5%) failed. Success group: the most negative fluid balance, APACHE II in 20 (14-25), weak cough in 58 (13.9%). Failure group: the most positive fluid balance, APACHE II in 23 (19-29), weak cough in 31 (47.7%), abundant amount of pulmonary secretions in 47.7%.

**Conclusion:**

positive fluid balance and the presence of inefficient cough or inability to clear the airway were predictors of extubation failure.

## Resumo

**Objetivo:**

investigar os fatores associados à falha de extubação de pacientes na unidade de terapia intensiva.

**Método:**

caso-controle não pareado, longitudinal, retrospectivo e quantitativo com a participação de 480 pacientes por meio de parâmetros clínicos para desmame ventilatório. Dados analisados por: Teste Exato de Fisher ou o teste Qui-quadrado; teste t de Student bicaudal não pareado; e teste de Mann-Whitney. Admitiram-se significantes valores de P menores ou iguais a 0,05.

**Resultados:**

dos pacientes, 415 (86,5%) tiveram sucesso e 65 (13,5%) falharam. Grupo sucesso: balanço hídrico mais negativo, APACHE II em 20 (14-25), tosse fraca em 58 (13,9%). Grupo falha: balanço hídrico mais positivo, APACHE II em 23 (19-29), tosse fraca em 31 (47,7 %), quantidade abundante de secreção pulmonar em 47,7 %.

**Conclusão:**

o balanço hídrico positivo e a presença de tosse ineficiente ou incapacidade de higienizar a via aérea foram preditores de falhas de extubação.

## Resumen

**Objetivo:**

investigar los factores asociados al fracaso de la extubación de pacientes en la unidad de cuidados intensivos.

**Método:**

caso y control no apareado, longitudinal, retrospectivo y cuantitativo con la participación de 480 pacientes mediante parámetros clínicos para el destete de la ventilación. Datos analizados por: Prueba Exacta de Fisher o prueba de Chi-cuadrado; prueba t de Student de dos colas para datos no apareados; y prueba de Mann-Whitney. Se admitieron valores de P significativos menores o iguales a 0,05.

**Resultados:**

de los pacientes, 415 (86,5%) tuvieron éxito y 65 (13,5%) fracasaron. Grupo de éxito: balance hídrico más negativo, APACHE II en 20 (14-25), tos débil en 58 (13,9%). Grupo de fracaso: balance de líquidos más positivo, APACHE II en 23 (19-29), tos débil en 31 (47,7%), abundante cantidad de secreciones pulmonares en 47,7%.

**Conclusión:**

el balance hídrico positivo y la presencia de tos ineficaz o incapacidad para higienizar la vía aérea fueron predictores de fracaso de la extubación.

**Highlights**

(1) For orotracheal extubation, the Spontaneous Breathing Test must be performed.

(2) Positive fluid balance was a predictor of extubation failure.

(3) Inefficient coughing or inability to clear the airway was a predictor of failure.

(4) Need for objective assessment of fluid balance and cough.

## Introduction

In the United States, 33% of patients admitted to an intensive care unit (ICU) receive invasive mechanical ventilation (IMV) and approximately 15 million surgical procedures requiring orotracheal intubation are performed^1^. In addition, approximately 650,000 emergency intubations not related to surgical procedures are performed^2^. As for national data, around 40% of patients admitted to the ICU are intubated and require IMV^3^. 

Although the processes of orotracheal intubation (OTI) and IMV cause apprehension in the multiprofessional team and in the family members because they refer to the worsening of the patient’s clinical condition, this therapeutic approach is vital for the maintenance of respiratory capacity in the critical period during the treatment of the underlying disease^4^. The IMV patient release process consists of three phases: the spontaneous breathing readiness test that determines whether the patient has respiratory drive; weaning where the ventilator’s degree of ventilatory support is gradually reduced over a period of time; and removal of the endotracheal tube^5^. Removing the patient from IMV is called extubation and consists of removing the ventilatory prosthesis called orotracheal tube (OTT)^6^.

Although it is an important life support, the prolonged use of IMV causes complications related to the positive intrathoracic pressure generated by the MV, oxygen toxicity and consequences of sedation^7^. It is known that these complications increase the mortality rate, justifying the need to remove IMV as early as possible^8^. In addition to the complications associated with prolonged IMV use, extubation failure also results in longer ICU stays, increased risk of pulmonary infection, need for tracheostomy, and an increased mortality rate between 23.5% and 53%^9^ ^-^ ^12^.

In order to make ventilator weaning and IMV discontinuation occur at the optimal time, The American College of Chest Physicians and the American Thoracic Society recommend the use of spontaneous mode (PSV) during Spontaneous Breathing Test (SBT) with pressures inspiratory measures around 5 to 8 cmH_2_O and protocols for daily interruption of sedation^10^ ^,^ ^13^. The act of performing SBT before interrupting IMV can help to avoid extubation failures related to ventilatory incapacity^12^. In addition, the work of breathing immediately after extubation is significantly higher than during the use of the orotracheal tube and persists for approximately 24 hours, as edema may occur in the airways near the glottis, responsible for the increase in post-extubation work^14^.

Extubation is considered when the endolaryngeal prosthesis is removed without the need for reintubation within the next 48 hours or within seven days^15^. The extubation failure rate increases when the success assessment time is taken into account and is between 12.5%, 15.3% and 22% in patients assessed at 24 hours, 72 hours and more than 72 hours respectively, being 26% the mean rate assessed 48 hours after extubation^16^.

The literature indicates other clinical variables that are associated with the risk of extubation failure, such as poor blood gas control^17^, rapid and shallow breathing index (RSBI) >105 breaths/L/min^11^ ^,^ ^15^, muscle weakness acquired in ICU^8^, prolonged stay on IMV (>7 days)^8^ ^,^ ^12^ ^,^ ^16^, ineffective cough^12^ ^,^ ^14^ ^-^ ^15^, bronchial hypersecretion^18^ ^-^ ^19^, positive fluid balance^14^ and critical condition high^16^ ^,^ ^18^.

Thus, a larger study showed that all clinical predictors have low predictive value when analyzed individually, suggesting a multifactorial assessment^20^. In this context, the objective was to investigate the factors associated with the failure of orotracheal extubation of patients in the intensive care unit.

## Method

### Study design

Unpaired, longitudinal, retrospective and quantitative approach case-control^21^.

### Setting

Data collection took place in a teaching hospital, of special size, in the countryside of Sao Paulo, a care reference in medium and high complexity for 102 cities with 706 beds, of which 114 were ICU. 

### Period

Data collection took place between January 2019 and December 2020.

### Population

**Figure 1:**
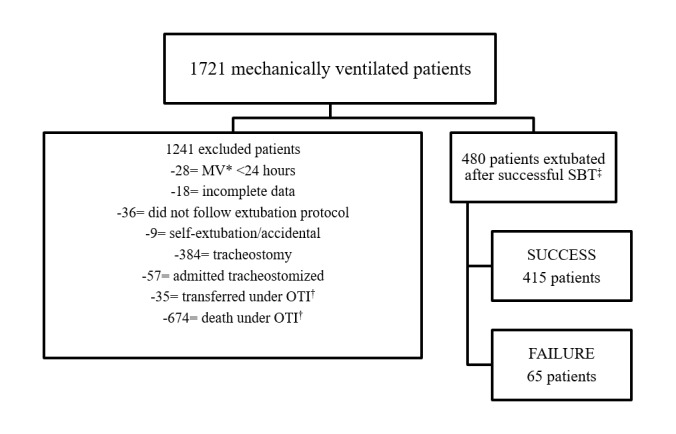
Flowchart of patients admitted to the ICU undergoing IMV and criteria for inclusion in the research

Data were collected in two ICUs, totaling 37 beds. Of a total of 1721 patients under IMV, 480 were extubated following the criteria established by the safe extubation protocol. (Figure 1).

*Mechanical ventilation; ^†^Orotracheal intubation; ^‡^Spontaneous Breathing Test

### Selection criteria

We included a total of 480 patients over 18 years old of both genders, submitted to IMV for a period longer than 24 hours, extubated in a planned way, respecting the Brazilian Guidelines on Mechanical Ventilation, which determine the performance of SBT under the following conditions: cause of respiratory failure must be solved or controlled; Arterial Oxygen Pressure (PaO_2_) greater than 60 mmHg with Inspired Oxygen Fraction (FiO_2_) less than or equal to 40% and Positive End-Expiration Pressure (PEEP) between 5 and 8 cmH_2_0; hemodynamic stability; good peripheral perfusion; normal or falling lactate; absence of coronary insufficiency or cardiac arrhythmias with hemodynamic repercussions; adequate nutrition; absence of excessive intravascular volume on the day before SBT; and presence of acid-base balance, using the T-Tube or the PSV modality for a period corresponding to 30 to 120 minutes^6^.

In the T-Tube test, the OTI was disconnected from the MV and attached to an oxygen source, breathing without any pressure support. Another way to perform SBT is using the PSV modality, through which minimum parameters of care are offered, proving that there is no longer dependence on MV^4^ ^,^ ^6^ ^,^ ^22^ and that there is no difference between SBT strategies in ventilatory weaning^23^. Extubation success is considered when the endolaryngeal prosthesis is removed without the need for reintubation within the next 48 hours or up to seven days^4^ ^,^ ^12^ ^,^ ^22^.

In the case of failures, the decision to reintubate the patient was made by the medical team according to the following symptoms: tachypnea, tachycardia, hypertension, hypoxemia, acid-base imbalance, cardiac arrhythmia, agitation or altered level of consciousness, sweating and evident increase in breathing work.

### Study variables

The data from the medical records were recorded in an Excel spreadsheet. The variables were the tests that preceded extubation: PaO_2_ with normal values between 80 and 100 mmHg, PaCO_2_ between 35 and 45 mmHg, hemoglobin with normal values between 12.5 and 16.5 g/dL in men and 11.5 and 15.5 in women^24^.

Arterial blood gases were collected with an inspired oxygen fraction of 30% to 40%. The time of IMV before extubation was also verified. The variables collected from the daily medical report on the day of extubation were: total fluid balance, which is calculated through the difference between the volume infused and the volume eliminated, referring to the amount accumulated during hospitalization up to 24 hours prior to the extubation procedure; and the evaluations of the clinical picture and criticality with APACHE II and SOFA^17^ ^,^ ^19^ ^,^ ^25^.

Regarding the amount of pulmonary secretion, this was verified in the physical therapists’ notes of the last 24 hours before extubation, being defined as a large amount of secretion when the airway aspiration procedure was performed according to the patient’s need in a period of less than two hours between aspirations^22^. The patient was evaluated for cough effectiveness by the physical therapist by observing the contraction of the abdominal muscles associated with the ability to mobilize secretion by the OTT^26^.

In this study, coughing was defined as effective when the patient was able to eliminate bronchial secretions after extubation, and considered ineffective when orotracheal aspiration was required due to inability to clean the airway/eliminate bronchial secretions. This assessment took place within a period of up to 24 hours after extubation, described in the notes of the physiotherapy and nursing teams. The dependent variable or primary outcome was the success or failure of extubation determined after 48 hours of extubation.

### Data analysis

Descriptive analysis was performed using mean and standard deviation, median and interquartile range, count and percentage. In the comparative analyses, Fisher’s exact test or chi-square test for qualitative variables, unpaired two-tailed Student’s t test and the Mann-Whitney test were used. The statistical program STATSDIRECT 3.3.5 was used. An alpha error of 5% was admitted, and P values less than or equal to 0.05 were considered significant. In the multivariate analysis, using Logistic Regression, it was possible to simultaneously analyze the multiple variables with all those that presented values of P<0.10 in the univariate analysis. Odds ratio was used as a measure of association in case-control studies.

### Ethical aspects

The research complied with the recommendations of Resolution No. 466/12 of the National Health Council on Research Involving Human Beings. Opinion: 3 764 211.

## Results

Of 480 patients extubated following the institutional protocol, 415 (86.5%) were successful after 48 hours and 65 (13.5%) failed, requiring reintubation and return to IMV. Women had a higher incidence of failure and a more positive fluid balance 622.5 ml (-324.5-1997.5) compared to men 246 ml (-758.5-1682.1) (p=0.0297). The characterizations and clinical variables of the patients are shown in Table 1.

**Table 1:** Characterization and clinical variables of extubated patients as established in the Brazilian guidelines for mechanical ventilation. São José do Rio Preto, SP, Brazil, 2022

	Total (n=480)	Success (n=415)	Failure (n=65)	p value
Age, years, mean (sd*)	55.5 (17.4)	55.1 (17.1)	55.9 (17.7)	0.7517^‡^
Male, n (%)	282 (58.7)	254 (90)	28 (10)	0.0067^§^
Female, n (%)	198 (41.3)	161 (81.3)	37 (18.7)	
*Inpatient unit*				
ICU A,n (%)	279 (58.1)	246 (88.1)	33 (11.9)	0.2476^§^
ICU B, n (%)	201 (41.9)	169 (84.1)	32 (15.9)	
*Staff*				
Surgery, n (%)	207 (43.1)	179 (86.5)	28 (13.5)	>0.9999^§^
Clinical, n (%)	273 (56.9)	236 (86.4)	37 (13.6)	
Days Orotracheal intubation, median (IQR^†^)	4 (2-5)	4 (2-5)	5 (3-5)	*0.0132* ^(c)^
PaO_2_, mmHg, median (IQR)	109 (90-136.25)	110.1 (91-137)	101.4(79-127)	*0.0324* ^||^
PaCO_2_, mm\hg, median (IQR)	37 (33-43)	37.4 (32.5-42)	39.2 (34-46)	0.0702^||^
Hemoglobin, g/dL, mean (sd)	9.8 (1.9)	10 (2.1)	9.7(1.7)	0.2042^‡^
Hydric balance, ml, median (IQR^†^)	1226 (2704)	397 (-653-1654.5)	1150 (144-3025)	0.0003^||^
Weak cough n (%)	89/480 (18.5)	58/415 (13.9)	31/65 (47.7)	< 0.0001^§^
Copious secretion, n (%)	237/480 (49.3)	197/415(47.4)	40/65 (61.5)	0.0448^§^
APACHE II/Emergency,	20 (14-26)	20 (14-25)	23 (19-29)	*0.018* ^||^
SOFA/General	6 (4-8)	6 (4-8)	6 (4.75-8)	0.7403^||^

*sd = Standard deviation; ^†^IQR = Inter-quartile range; ^‡^Unpaired Two-tailed Student T Test; ^§^Fisher’s Exact Test; ^||^Mann-Whitney Test

The Logistic Regression that allowed establishing the chance of failure was: logit FAILURE = -2.051638, being +0.000165 Water Balance; and +1.923026 Cough strength. The coefficients that accompany the independent variables indicated the importance of each variable for the occurrence of the event, as shown in Table 2.

**Table 2:** Multivariate analysis - logistic regression of factors associated with extubation failure. São José do Rio Preto, SP, Brazil, 2022

Parameter	Odds Ratio (OR)	95% Confidence Interval	p
Hydric balance	1.000.169	1.000061 a 1.000277	0.0026
Ineffective cough	6.588.996	3.566588 a 12.172661	<0.0001

Of the patients who evolved with extubation failure, 39 (60%) underwent tracheostomy and of the 65 who failed, 38 (58.4%) died during hospitalization. According to Table 3, the stratification of patients who failed according to fluid balance is evident.

**Table 3:** Distribution of patients who had extubation failure in relation to fluid balance. São José do Rio Preto, SP, Brazil, 2022

	Total extubated patients (n)	Patients with failed extubation (n)	Extubation failure rate (%)
HB* <-2000 ml	44	4	9.09
HB* -1999 ml up to -1000 ml	43	2	4.65
HB* -999 ml up to 0 ml	100	7	7
HB* 1 ml up to 1000 ml	111	18	16.22
HB* 1001 ml up to 2000 ml	68	12	17.65
HB* >2001 ml	114	22	22

*HB = Hydric balance

## Discussion

Among the indicators used to prevent extubation failure, positive fluid balance and inefficient coughing with the presence of abundant pulmonary secretions were the most strongly associated with extubation failures in critically ill patients in the ICU. Considering the relevance of orotracheal extubation for the patient, this moment needs to be very well evaluated and performed correctly. Although the variables are favorable to the safe extubation procedure, some patients may still have failures.

In 2020, in a systematic review with a total of 10 randomized controlled studies involving 3165 patients, the incidence rate of unfavorable evolution after orotracheal extubation was 26%^23^. In 2019, one study presented a failure rate between 12.5% and 22%^22^, and another one, around 14%^17^, reaching a failure rate of 36% in those with severe ICU-acquired muscle weakness^12^.

A study reveals as an ideal failure rate the value between 5% and 10%, taking into account the age, comorbidity and severity of the patient, in addition to the evaluation in the domains: clinical, nutritional, neurological, hydro electrolytic, and also cough strength and ability to protect the airway^27^. In this study, among the 480 extubated patients, 415 (86.5%) evolved favorably, with success, and failure occurred in 65 (13.5%) who required a return to ventilator prosthesis within 48 hours after extubation, which is in agreement with the rates found in the literature.

It is known that airway compromise occurs due to the presence of the orotracheal tube due to mucosal injury or granulation tissue caused by direct pressure from the tube. In addition, the moment of intubation can cause lesions in the presence of a difficult airway, with symptomatic repercussions at the time of extubation^11^ ^,^ ^28^. Factors such as the use of large-caliber tubes, high cuff pressure or friction between the tube and the cuff along the airway caused by constant neck movements determine an inflammatory process at the site^29^.

A determining variable for extubation failure is the period in which the patient is intubated. In this study, the failure group had a greater number of days of intubation, demonstrating that shorter IMV time was related to higher extubation success rates, showing that failure due to structural injury and airway instability was probably determined by the duration of IMV, as this increased time generally compromises the viability of the tracheal mucosa^29^.

Corroborating this study, a recent multinational prospective study with a total of 2729 patients showed that the patients with short IMV duration had a reintubation rate of 1.3%, while those with a longer duration had a rate of 45.4%^8^.

Failure of orotracheal extubation due to respiratory failure occurs when there are changes in oxygenation or ventilation levels^30^. To perform extubation, the results of arterial blood gas analysis are taken into account, PaO_2_ levels above 80 are expected with 40% FiO_2_ and PCO_2_ levels lower than or equal to 44 mmHg^22^ ^,^ ^31^ ^-^ ^32^.

In this study, respiratory failure was found to be the most frequent cause of failure, which occurred in 29 patients of the 65 who failed (44.6%). However, when evaluated, arterial blood gas values were within the normal range, and PaO_2_ of extubation failure caused by respiratory failure did not differ from the values of the other causes of failure. Probably, other factors were associated to determine the failure, such as the presence of hypervolemia, since gas exchange was impaired by the removal of positive interalveolar pressure or muscle weakness, with difficulty in expectoration.

The second most prevalent cause of extubation failure in this study was glottic edema, which occurred in 15 of the 65 patients who failed (23.1%). The pathophysiology of glottic edema is determined by the presence of the endotracheal tube with pressure on the posterior wall of the pharynx, inducing edema and ulceration, which occur, on average, in 9.4% of extubated patients^33^. However, the occurrence was in only 3.1% of extubated patients. A probable explanation for the low incidence of glottis edema may be related to the maintenance of ideal values of cuff pressure, since the control and checking of cuff pressure are carried out in all nurses’ work shifts.

One way to try to reduce failure due to edema of the glottis indicated by the French Society of Anesthesia and Intensive Care in 2019 is to perform the cuff test, as it indicates the presence of post-extubation laryngeal stridor resulting from spasm of the laryngeal area due to an inflammatory process^34^ ^-^ ^35^.

A data observed in the medical records, but not analyzed in this study, refers to the fact that those who failed due to glottic edema had an inappropriate awakening with psychomotor agitation (hyperactive delirium) and excessive movement of the head, and also probable tracheal mucosal injury. The level of consciousness and degree of anxiety seem to have an impact on the process of weaning from ventilation and extubation, since very agitated patients, in addition to “injuring” the tracheal mucosa, need doses of sedatives or tranquilizers, and the level of consciousness may fluctuate after removal of the ventilatory prosthesis with probable deficit in the protection of the airways^12^.

Another factor related to ventilation weaning and the peri-extubation period is hemoglobin, which when at lower levels (less than 10.0 g/dL), predicts extubation failure, as it reduces the ability to carry oxygen, compromising the aerobic metabolism of the muscles and resulting in respiratory failure^36^. In this study, values close to ideal were obtained, showing that extubation occurred with safe hemoglobin levels.

It is known that extubation success is related to fluid balance, and excessive intravascular volume should be avoided during the 24 hours prior to the procedure and the use of diuretics should be considered if it is not contraindicated^4^, with importance for blood volume restriction in the 48-72 hours after extubation^37^. However, values determined for the fluid balance are not established in the institution’s safe extubation protocol.

In this study, hypervolemic patients failed extubation, probably due to the fact that hypervolemia led to pulmonary congestion and consequent impairment in the diffusion process through the alveolar-capillary membrane, leading to hypoxemia and increased pulmonary ventilatory work^14^. Negative or balanced fluid balance determined extubation failure rate around 7% and positive fluid balance determined a rate higher than 16%, reaching 22% when the balance was positive and greater than 2000 ml. It was also evidenced that the female gender had a higher incidence of extubation failure, which could probably be related to the positive fluid balance.

Another important characteristic in the post-extubation evolution is generalized muscle strength and respiratory muscle strength, as patients with muscle weakness have higher extubation failure rates^12^ ^,^ ^38^. However, extubation should not be delayed in patients with adequate cough, despite presenting with peripheral weakness^12^. Related to muscle strength after successful SBT, the most important parameter for successful extubation is the cough efficiency for the management of bronchial secretion, as an effective cough is essential to clear secretions from the more proximal airways^9^ ^,^ ^15^ ^,^ ^18^ ^-^ ^19^ ^,^ ^26^, with an extubation failure rate of about 33-41% in patients with ineffective cough, compared to 5-8% in those with effective cough strength^12^ ^,^ ^39^.

Associated with the presence of efficient coughing with the elimination of bronchial secretion, the integrity of the pharyngeal muscles reduces the occurrence of extubation failure. Lesions or alterations in the neuromuscular tone of this region result in swallowing disorders with consequent inability to protect the airway by swallowing secretions after coughing or even saliva, resulting in the perception of excessive pharyngeal secretions(40) and consequent need for frequent orotracheal aspiration, which constitutes a risk factor for extubation failure^4^.

As for the cough strength considered weak, it was found in 47.7% of the failure group, showing a high incidence of weak cough related to extubation failure. This research suggests that the cough strength evaluated as effective by the professional, subjectively and before extubation, was wrong, since many did not have a satisfactory cough after extubation.

Another factor considered was the degree of criticality of the patient in relation to the outcome of extubation, and the APACHE II score showed that severity impacts the result of extubation.

The length of stay in the ICU and mortality are increased in patients who fail, with mortality ranging from 23.5% to 53%^9^ ^-^ ^12^ ^,^ ^18^. In this study, 38 (58.5%) of the 65 patients who failed died during hospitalization, with an impact on mortality in the population with an unfavorable outcome of the extubation process. In view of these considerations, extubation is a step that must be performed with the most complete evaluation possible, aiming at success, especially when mortality data and hospital costs are evaluated.

In 2019, the French intubation and extubation guideline determined that if the patient, after approval in SBT, presents any risk for extubation failure, his extubation should be postponed. Therefore, the decision to extubate follows an established protocol. However, it is possible to reassess extubation until the patient shows improvement in some of the variables^34^. There is also guidance for extubation to be postponed if risk factors for extubation failure can be corrected within three days^39^.

According to the literature and considering the results, with cough efficiency as the main factor associated with extubation failure, this study suggests a careful assessment of the patient’s ability to cough in an objective manner, with the proposal of PCF (Peak Cough Flow) greater than 60 L/min evaluated in the IMV, and also the management of blood volume in view of the preparation for extubation and in the post-extubation period, since hypervolemic patients present pulmonary congestion. Considering the complexity of health care, the important thing is to expand assessment strategies during this moment considered so decisive for the conduction of the treatment plan for critically ill patients.

The limitations of this study are due to the fact that, as it is an observational study, in which it is not possible to demonstrate a causal relationship between the associated factors and extubation failure, as well as the fact that the study was carried out in a single teaching hospital, of special size, it can become difficult to generalize the results found, and also due to the subjectivity in the evaluation of the cough force perceived by each professional to perform the extubation.

## Conclusion

Among the factors associated with extubation failures, positive fluid balance and the presence of inefficient coughing or inability to clear the airway were predictors of reintubation. The implication for clinical practice was focused on the question of confirming the need to stratify and evaluate these parameters before extubating the critically ill patient in the intensive care unit, in addition to considering the period of orotracheal intubation greater than five days, APACHE II score greater than 23 and the abundant amount of bronchial secretion.

The contribution made by this study was to confirm the importance of using the safe extubation protocol and to point out the parameters that serve as predictors for failures, in order to provide greater safety to the patient intubated in the ICU.
